# Music-induced physiological markers for detecting Alzheimer's disease using machine learning

**DOI:** 10.3389/fnagi.2025.1701970

**Published:** 2025-11-24

**Authors:** Rodrigo Lima, Gonçalo Barradas, Sergi Bermúdez i Badia

**Affiliations:** 1 Faculdade de Ciências Exatas e da Engenharia, Universidade da Madeira, Funchal, Portugal; 2 Agência Regional para o Desenvolvimento da Investigação, Tecnologia e Inovação, Funchal, Portugal; 3 NOVA Laboratory for Computer Science and Informatics, Universidade Nova de Lisboa, Lisboa, Portugal; 4 School of Health and Society, University of Salford, Salford, United Kingdom

**Keywords:** Alzheimer's disease, dementia, electrodermal activity, electromyography, emotional responses, machine learning, music

## Abstract

**Introduction:**

Alzheimer's disease (AD) is characterized by progressive cognitive and emotional decline, highlighting the need for novel, non-invasive biomarkers to aid in early detection, monitoring, and stage-specific interventions. This study investigates music-evoked physiological responses as potential biomarkers of AD and evaluates their translational value using machine learning (ML).

**Materials and methods:**

A total of 36 AD patients, spanning different severity levels, listened to emotionally evocative musical excerpts while electrodermal activity and facial electromyography (corrugator and zygomaticus muscles) were recorded. Machine learning models were then trained on these signals to classify the presence and severity of AD and to detect residual emotion-specific physiological responses elicited by music.

**Results:**

Physiological reactivity to music declined with disease progression, with positive emotions eliciting more distinct responses than negative ones. The Random Forest classifier distinguished AD patients from healthy controls with 70.5% accuracy, while the Naïve Bayes model predicted severity with 65.6% accuracy, demonstrating that ML models can detect subtle music-evoked physiological differences even in individuals with AD.

**Discussion:**

Music-evoked physiological signals reflect the hierarchical disruption of emotion-related neural circuits in AD and hold promise as complementary biomarkers for disease presence and stage. When combined with machine learning (ML), these measures provide a non-invasive, ecologically valid approach to support early detection, monitoring, and the development of stage-specific interventions.

## 1 Introduction

Dementia is a progressive neurodegenerative disorder characterized by a gradual decline in cognitive functions, including memory, learning, orientation, language, and judgment. Alzheimer's disease (AD) accounts for 60%–80% of all dementia cases and often begins long before symptoms become apparent, with progression varying among individuals (Matziorinis and Koelsch, 2022; Ferri et al., 2009). Although pharmacological treatments can alleviate some symptoms, their efficacy is limited and often associated with adverse side effects (Wollen, 2010; Barradas et al., 2021), leading to an increased emphasis on non-pharmacological interventions such as music therapy (Matziorinis and Koelsch, 2022).

Research shows that individuals with AD respond positively to music, even in advanced stages, with engagement linked to improvements in mood, behavior, and cognitive performance (Cuddy et al., 2012; Sakamoto et al., 2013; Narme et al., 2014; Särkämö, 2018). Musical abilities, particularly memory for familiar tunes, often remain relatively preserved in AD (Warren et al., 2003), highlighting music as a potential therapeutic tool. Music can trigger memories and feelings, elicit strong emotions, and promote connections with oneself and loved ones (Matziorinis and Koelsch, 2022). However, some studies suggest that while AD patients may struggle to identify specific emotional categories in general social contexts (e.g., facial expressions), emotional responses to music—reflected in arousal and valence—are often preserved (Gosselin et al., 2005; Cuddy et al., 2015). This distinction supports the use of music as both a therapeutic and diagnostic tool for assessing residual emotional processing in AD.

In recent years, several clinical studies have demonstrated that music-based interventions can serve not only as therapeutic modalities but also as potential tools for early detection and monitoring of AD. For instance, Mangiacotti et al. (2024) developed and validated the 15-min Music Cognitive Test (MCT), which reliably discriminates mild cognitive impairment and early AD from healthy controls by assessing phonological, rhythmic, and melodic encoding processes. In a related trial, the same group reported that a tailored music therapy program slowed the decline in episodic memory in mild-to-moderate AD patients compared to a no-music control group, with effect sizes comparable to those of standard pharmacotherapies. Neuroimaging evidence further supports music's diagnostic and monitoring potential. The Alzheimer's and Music Therapy (ALMUTH) trial utilized repeated fMRI and diffusion-tensor imaging over 12 months to demonstrate that active singing interventions were associated with a reduced brain-age gap estimation and enhanced hippocampal activation, which predicted cognitive trajectories in prodromal Alzheimer's disease (Flo et al., 2022).

Neuropathologically, AD is defined by extracellular amyloid-beta (*Aβ*) deposition, intracellular tau neurofibrillary tangles, and subsequent neuronal degeneration (Mauldin, 2013; Serrano-Pozo et al., 2011). Initial damage typically affects the hippocampal pathway–including the entorhinal cortex, hippocampus, and posterior cingulate cortex–while primary sensory and motor regions are spared in early stages (Frisoni et al., 2010; Cuingnet et al., 2011; Villain et al., 2012; Lehmann et al., 2013). Musical memory, however, appears less affected by cortical degeneration (Cuddy et al., 2015; Jacobsen et al., 2015), suggesting that music may help mitigate cognitive and emotional deficits associated with AD. Despite widespread cortical atrophy, regions within the limbic system, such as the amygdala and anterior cingulate cortex, often remain relatively preserved in the early and moderate stages of AD (Jacobsen et al., 2015; Frisoni et al., 2010). These structures are central to emotional processing and are strongly recruited during music listening, supporting emotional memory, reward, and autonomic regulation (Koelsch, 2020; Salimpoor et al., 2011). Thus, even when higher-order cognitive networks deteriorate, music can engage residual emotional and reward-related circuits, potentially alleviating affective and behavioral symptoms while providing access to autobiographical memory and a preserved sense of self.

Mechanisms underlying the benefits of music therapy include neurogenesis and neuroplasticity, as music-evoked emotions activate the hippocampus and support synaptic formation, thereby enhancing memory and mood (Savage et al., 2021; Koelsch, 2020). Music also promotes dopamine release, engages the brain's reward system, and counteracts age-related cognitive decline (Salimpoor et al., 2011). Moreover, by modulating inflammatory and autonomic processes, music can exert neuroprotective effects through reduced stress and immune activation (Kinney et al., 2018). Collectively, these mechanisms demonstrate how music engages distributed neural networks spanning limbic, reward, and autonomic systems to support cognitive and emotional functioning in AD.

The BRECVEMA framework explains music-evoked emotional responses through eight mechanisms: Brain stem reflex, Rhythmic entrainment, Evaluative conditioning, Contagion, Visual imagery, Episodic memory, Musical expectancy, and Aesthetic judgment (Juslin, 2013). In this study, four mechanisms were targeted using validated musical excerpts: Brain stem reflex (surprise), Contagion (sadness), Episodic memory (happiness), and musical expectancy (anxiety), with a neutral control condition (Juslin et al., 2015).

Building on these findings, music-based biomarkers are emerging as promising tools for the early detection and monitoring of AD. Recent reviews have highlighted the growing importance of early biomarkers for detecting AD progression, including molecular, neuroimaging, and cognitive markers (Prajapati et al., 2024). Complementing these approaches, music-evoked physiological signals such as EDA and EMG provide non-invasive, real-time measures of emotional and cognitive reactivity that may serve as translational biomarkers. For example, the ADMarker project exemplifies the potential of combining music-evoked physiological responses with machine learning. This multimodal federated learning system integrates various digital biomarkers derived from music-based interventions to monitor AD progression in natural living environments. The system demonstrated up to 93.8% accuracy in detecting a comprehensive set of digital biomarkers and 88.9% accuracy in identifying early AD (Ouyang et al., 2024).

Assessing music-evoked responses in AD populations presents challenges, as emotional reactions are difficult to quantify due to impaired self-reporting and reduced facial emotion recognition (Koff et al., 1999; Albert et al., 1991; Fangmeng et al., 2018). Music-evoked physiological signals offer an objective and non-invasive means to overcome these limitations. Given that both emotional valence and autonomic arousal are mediated by networks that remain partially functional in early AD, electrodermal activity (EDA) and facial electromyography (EMG) provide accessible physiological proxies of residual emotional network engagement during music listening. Facial EMG measures the activity of the corrugator and zygomaticus muscles to capture emotional valence. In contrast, EDA reflects sympathetic arousal, providing real-time monitoring even in the absence of overt expressions (Cacioppo et al., 2007; Künecke et al., 2014; Lima et al., 2024; Boucsein, 2012). EDA and EMG responses reflect the activation of the central emotion-related circuits. EDA is primarily modulated by amygdala-hypothalamic pathways and insular pathways within the salience network that regulate sympathetic arousal. In contrast, facial EMG activity is influenced by cortico-subcortical circuits involving the motor cortex and basal ganglia, which mediate valence and reward (Cacioppo et al., 2007; Boucsein, 2012). Because these systems are partly preserved in early AD, music-evoked physiological reactivity provides a peripheral index of residual emotional and autonomic network engagement. Therefore, these physiological signals can serve as music-based biomarkers, providing insights into alterations in emotional processing in AD and enabling translational applications for early detection, disease monitoring, and personalized therapeutic interventions.

Machine learning (ML) has emerged as a powerful tool for AD diagnosis, prognosis, and classification. Traditional clinical assessments are time-consuming and may fail to detect early-stage disease (Zhu et al., 2020). ML models, including support vector machines, random forests, and deep learning architectures, have demonstrated high accuracy in analyzing neuroimaging, clinical, and neuropsychological data (So et al., 2017; Mirzaei and Adeli, 2022; Mathkunti and Rangaswamy, 2020). Beyond diagnosis, ML can predict disease progression and patient outcomes (Zhang J. et al., 2024; Qiu et al., 2020), while interpretable models enhance clinical applicability by identifying relevant brain regions and biomarkers. Despite these advances, no studies have yet leveraged peripheral physiological signals, such as EDA and EMG, to predict dementia or to assess emotional responses during music therapy.

This study aims to fill these gaps by evaluating emotional responses to music in AD patients across different severity levels using EDA and facial EMG and by exploring the feasibility of ML models to classify emotional content, distinguish AD patients from healthy controls, and differentiate between disease severity stages.

## 2 Methodology

### 2.1 Participants

Participants were recruited from a healthcare facility in Madeira through a formal collaboration between ARDITI/University of Madeira and the institution. The study was reviewed and approved by the institution's clinical board. Patient recruitment was overseen by the board, which selected individuals who were diagnostically assessed by a team of physicians and nurses and diagnosed with possible or probable AD, with updates on participant availability provided.

The following exclusion criteria were used to select AD patients: no history of head trauma, stroke, alcoholism, or known hearing problems.

The study included 36 participants, all native Portuguese speakers, with an average age of 77 ± 5.07 years. All patients were taking anti-dementia medication at the time of the study. Cognitive function was assessed and categorized into three groups based on AD severity, according to the Mini-Mental State Examination (MMSE): The Mild group consisted of 12 participants (eight females, four males), with an average age of 77.25 ± 5.39 years (range 70–88), diagnosed with possible or probable AD. The Moderate group included 12 participants (five females, seven males), with an average age of 76.16 ± 5.55 years (range 67–86). The Severe group comprised 12 participants (seven females, five males), with an average age of 77.58 ± 3.57 years (range 71–84).

### 2.2 Materials

#### 2.2.1 Hardware and software

The hardware setup for this study included a laptop to collect all physiological signals from the BiosignalsPlus wearable device. For this study, EDA and facial EMG signals were recorded at 16-bit resolution and 1,000 Hz. Furthermore, participants listened to the musical conditions through a pair of high-quality speakers (Creative Inspire T3300).

For software, OpenSignals was used to record and extract all physiological signals and to compute the features used in this study. The musical excerpts were presented using the free online platform eSurv (EUSurvey, 2021).

#### 2.2.2 Mini-Mental State Examination (MMSE)

The Mini-Mental State Examination is a validated and widely used tool for assessing cognitive function in research and clinical settings (Folstein et al., 1975). It includes five cognitive tests in the following domains: orientation, registration, attention and calculation, recall, and language (Arevalo-Rodriguez et al., 2021).

In this study, since our participants were all native Portuguese speakers, patients were assessed by the healthcare team using the adapted Portuguese version of the Mini-Mental State Examination (Guerreiro et al., 1994). The Mini-Mental State Examination has a total score of 30 points, with higher scores indicating better cognitive function, and it can be divided into the following cognitive impairment levels: Severe (0–9), Moderate (10–18), Mild (19–23), and No cognitive impairment (24–30).

### 2.3 Experimental procedure

The experiment was conducted over six weeks, with each participant participating in a session lasting approximately 45 min.

The study employed a mixed design, using a within-subjects approach to analyze the emotional content of music and a between-subjects approach to compare dementia severity levels. All participants listened to the same five musical excerpts, presented in a randomized order, each intended to evoke a specific target emotion: Happiness (Episodic Memory), Sadness (Contagion), Anxiety (Musical Expectancy), Surprise (Brain-stem reflex), and Neutral (Control). The musical stimuli used were previously validated by Barradas et al. (2021) in Portuguese elderly participants, including both healthy and AD patients. This validation confirmed that the target mechanism stimuli, originally developed in Swedish research contexts (Juslin et al., 2015), were also valid in the Portuguese cultural setting.

All music sessions were conducted individually in a quiet and familiar setting (the participant's room at the healthcare facility) by the researcher, who implemented the experimental protocol. An accredited healthcare professional supervised each session to ensure ethical compliance, participant safety, and adherence to the experimental protocol. Participants were informed that they would listen to music through high-quality loudspeakers, with sound levels kept consistent across all participants. Both the researcher and the professional ensured that participants remained engaged and attentive throughout the session.

Physiological sensors were then placed to measure EDA and facial EMG baseline levels. Facial EMG electrodes were placed on the left corrugator and zygomaticus muscles because these sites are well-established indicators of emotional valence, according to Cacioppo's guidelines (Cacioppo et al., 2007). The corrugator muscle activity increases in response to negative affect, reflecting frowning or distress, whereas zygomaticus activity increases during positive affect, reflecting smiling or pleasure. In contrast, EDA electrodes were placed on the palmar surface of the non-dominant hand at the thenar and hypothenar eminences to measure sympathetic arousal. The palmar surface is highly sensitive to changes in sweat gland activity, which reliably indexes autonomic nervous system engagement during emotional stimulation (Boucsein, 2012). Baseline recordings were collected while participants were in a relaxed, silent state.

Following this, participants listened to the musical excerpts. A short break was provided between each piece to allow physiological responses to return to baseline before the next stimulus was presented.

### 2.4 Biosignals processing

Physiological signals were recorded using the OpenSignals software. EDA was used to assess arousal levels, while facial EMG, recorded from the corrugator (negative valence) and zygomaticus (positive valence) muscles, evaluated emotional valence. Both EDA and EMG signals were automatically pre-processed by OpenSignals, including artifact removal and signal filtering. The software decomposed the raw EDA signal into tonic and phasic components. The average tonic component was computed for each musical excerpt and expressed in microSiemens (μS). EMG signals were filtered with a 6*th*-order Butterworth bandpass filter (28–250 Hz), and EMG activity was computed using the maximum voluntary contraction method. The average EMG level for each music condition was expressed in microvolts (μV). Signal quality was visually inspected by the researcher, and only segments corresponding to the musical excerpts were analyzed, thereby minimizing the risk of missing data. Feature extraction was limited to those available in the OpenSignals software add-on at the time of data collection.

### 2.5 Statistical analysis

Statistical analyses were conducted to examine differences in physiological responses across various emotional music content and to identify differences in physiological responses across levels of AD severity.

Data normality was assessed using the Shapiro–Wilk test. For within-subject comparisons of physiological responses across different emotional content of music, the nonparametric Friedman test was applied because the data were not normally distributed. *Post-hoc* pairwise comparisons were conducted using the Wilcoxon signed-rank test, with Bonferroni correction for multiple comparisons. An a priori power analysis (using G-Power v3.1.9.4) for the Friedman test, assuming a large effect size (*Cohen*′*s f* = 0.40), α = 0.05, target power (*power* = 0.80), and five within-subject conditions (five emotions), indicated a required sample size of nine participants.

For between-group comparisons (in terms of AD severity levels), the Shapiro–Wilk test was used again to assess normality. When data were normally distributed, a parametric one-way ANOVA was performed, followed by *post-hoc* *t*-tests with Bonferroni correction. If the data violated normality assumptions, the non-parametric Kruskal–Wallis test was used, with Mann–Whitney *U*-tests and the Bonferroni correction applied for *post-hoc* analysis. An a priori power analysis for the one-way ANOVA, assuming a large effect size (*Cohen*′*s f* = 0.40), α = 0.05, target power (*power* = 0.80), and three between-subject conditions (three severity groups), indicated a required sample size of 66 participants.

Due to the challenges of recruiting participants with specific stages of AD from a geographically constrained population, the final sample included 36 AD patients across three severity levels. While the a priori power analysis for the one-way ANOVA indicates that 66 participants would be required to detect large effects with 80% power, the current sample is sufficient to detect robust, large effects in physiological responses to emotional content while still providing exploratory insights into stage-specific physiological responses.

### 2.6 Machine learning

In this study, supervised ML models were employed to classify emotional content in music, distinguish between healthy and AD participants, and assess AD severity in AD patients using physiological signals. Supervised models learn from previously labeled data to classify new, unseen data by assigning each instance to its class (Lima et al., 2024).

#### 2.6.1 Models evaluated

The classifiers considered were as follows: K-Nearest Neighbors (KNN), SVM, Logistic Regression (LogReg), Naïve Bayes (NB), RF, and Neural Networks (NN)—Multi-Layer Perceptron (MLP). These classifiers were chosen based on their proven effectiveness and complementary strengths in classification tasks. KNN offers a simple, instance-based approach suitable for datasets with non-linear class boundaries. SVMs are robust classifiers that perform well in high-dimensional spaces and effectively handle non-linear relationships. LogReg provides a computationally efficient, interpretable baseline for binary classification problems. NB leverages probabilistic assumptions to deliver fast and often reliable results, especially when features are conditionally independent. RF is an ensemble method for handling noisy, high-dimensional data while reducing overfitting. Lastly, NNs capture complex nonlinear patterns across multiple layers, offering flexibility and powerful modeling capabilities. These models have all been widely used in related domains and provide a balanced spectrum of algorithmic complexity, interpretability, and predictive performance (Zhu et al., 2020; So et al., 2017; Vapnik, 1998; Breiman, 2001; Jain et al., 1996; Pedregosa et al., 2011; Gupta and Kahali, 2020).

#### 2.6.2 Hyperparameter selection

Hyperparameters for each model and classification task were optimized using a GridSearch procedure applied exclusively to the training data in each cross-validation fold. For each model, a predefined range of hyperparameter values was systematically evaluated to identify the parameters that maximized performance metrics on the training data while minimizing the risk of overfitting. The hyperparameters reported in each classification task correspond to the best-performing configurations found through this iterative search. This procedure ensures that the models were evaluated under consistent conditions and that the selected hyperparameters were determined systematically from the training data rather than chosen arbitrarily.

#### 2.6.3 Feature selection

Three features were extracted from each signal (EDA, EMG corrugator, and EMG zygomaticus): the average during baseline, the average during the musical excerpt, and the difference between these two measures. This feature processing produced a total of nine input features per sample. Due to the small number of features and their direct relevance to emotional arousal and valence, no additional feature selection was performed, and all features were included in the classification models.

#### 2.6.4 Cross-validation

Model evaluation was performed using the Leave-One-Subject-Out (LOSO) cross-validation method for all classification tasks. In each iteration, the data from one participant was held out as a testing set, while the data from the remaining participants was used for training. This procedure was repeated until every participant had been used as a testing set. Accuracy, Precision, Recall, and the F1-score were averaged across all folds to ensure robust performance estimates (Bishop, 2006; Mohammad and Nasir, Md, 2015).

#### 2.6.5 Classification of emotional content in music

Classification models were trained to predict the emotional content in music (anxiety, happiness, neutral, sadness, and surprise) from the participants' physiological signals.

Building on the study by Barradas et al. (2021), two datasets were used for this classification task, with each participant listening to all five musical excerpts: Dataset A consisted of 20 healthy participants and 20 AD patients (regardless of severity) from Barradas et al. (2021), used with permission from the authors. This dataset was used exclusively for this classification task, specifically to distinguish between healthy participants and AD patients, as described in the following section. Dataset B included 36 AD patients recruited for this study, as described in Section 2.1. This dataset was used to analyze physiological differences across emotional content and classify AD severity levels. Both datasets were collected using the same experimental protocol and were not merged to ensure consistency while avoiding potential biases that could arise from combining cohorts.

Data were normalized using a Standard Scaler, applied only to the training data to prevent data leakage and overfitting.

For Dataset A, models were trained and evaluated using a user-independent approach. The dataset was balanced, with each emotion equally represented in LOSO cross-validation. Hyperparameters were optimized via GridSearch, and the following configurations were used: KNN (algorithm = auto, n_neighbors = 4, weights = distance), SVM (gamma = auto, kernel = rbf, random_state = 0), LogReg (penalty = l2, random_state = 0, solver = lbfgs), NB, RF (bootstrap = True, criterion = gini, n_estimators = 100, random_state = 22), and NN (activation = logistic, random_state = 41, solver = lbfgs).

The same procedure was then applied to Dataset B, which consisted of 36 AD patients, balanced across their AD severity levels (12 mild, 12 moderate, and 12 severe). The same classifiers were evaluated in terms of performance using the LOSO, but with different hyperparameters: KNN (algorithm = auto, n_neighbors = 3, weights = uniform), SVM (gamma = scale, kernel = linear, random_state = 0), LogReg (penalty = l1, random_state = 14, solver = liblinear), NB, RF (bootstrap = True, criterion = entropy, n_estimators = 200, random_state = 31), and NN (activation = relu, random_state = 1, solver = lbfgs).

#### 2.6.6 Classification: healthy vs. AD participants

For distinguishing between healthy and AD participants, only Dataset A was used. The dataset included 20 participants per class (healthy vs. AD), providing a balanced binary classification setup. The same models were evaluated using LOSO, with the following configurations, obtained via GridSearch: KNN (algorithm = auto, n_neighbors = 4, weights = distance), SVM (gamma = scale, kernel = linear, random_state = 0), LogReg (penalty = l1, random_state = 39, solver = saga), NB, RF (bootstrap = True, criterion = entropy, n_estimators = 50, random_state = 36), and NN-MLP Classifier (activation = tanh, random_state = 19, solver = lbfgs).

#### 2.6.7 Classification of AD severity

Finally, for the classification of AD severity among patients in Dataset B, models were trained to distinguish Mild, Moderate, and Severe levels. Dataset B included 12 participants per severity level, ensuring a balanced three-class distribution. The same classification models were evaluated, with the following configurations, obtained via GridSearch: KNN (algorithm = auto, n_neighbors = 2, weights = uniform), SVM (gamma = auto, kernel = rbf, random_state = 0), LogReg (penalty = l2, random_state = 0, solver = lbfgs), NB, RF (bootstrap = True, criterion = entropy, n_estimators = 50, random_state = 29), and NN-MLP Classifier (activation = relu, random_state = 25, solver = lbfgs).

## 3 Results

This experiment presented musical excerpts with previously labeled emotions to AD patients, while their physiological signals (EDA and facial EMG) were recorded. Therefore, the goal was to evaluate whether these signals provided helpful insights regarding the arousal and valence of AD patients when listening to music. Moreover, machine learning models were trained and evaluated across three distinct tasks: predicting the emotional content of music, distinguishing between healthy and AD patients, and identifying severity levels within AD patients.

### 3.1 EDA and EMG differences to emotional content in music

Regarding the physiological response to emotional content in music, we tested for significant differences in EDA and facial EMG levels across different emotional contents, regardless of AD severity. This analysis focused exclusively on individuals with AD. No healthy control group was included in this analysis, as the aim was to assess emotional reactivity within the AD population from Dataset B. Comparisons with healthy participants were previously reported in Barradas et al. (2021) (Dataset A). In contrast, the present results are based exclusively on Dataset B.

**Figure 1:**
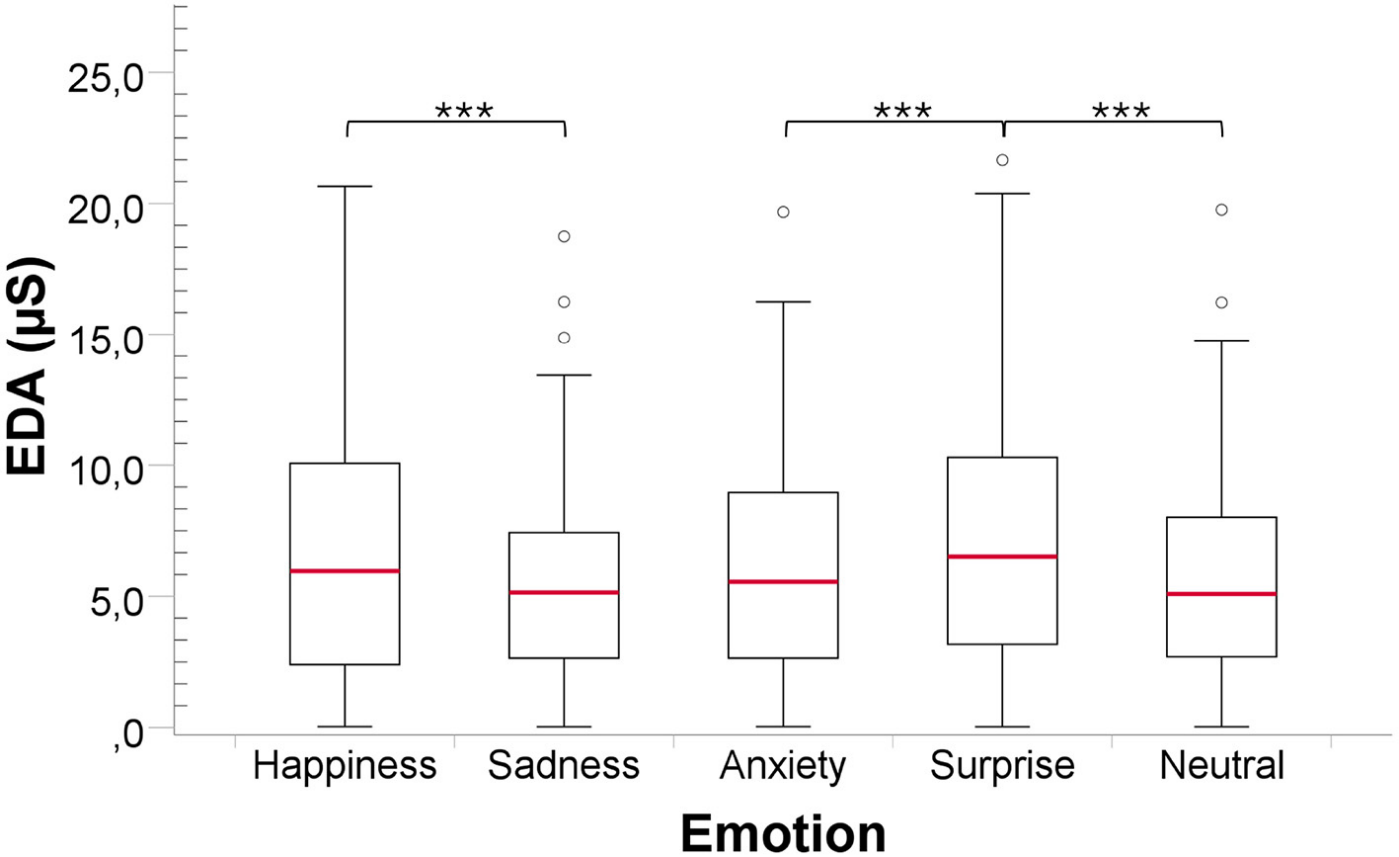
Comparison of EDA responses to the emotional content in music, for all participants regardless of AD severity. ***P_value_ < 0.001.

For EDA, the Friedman test revealed a significant difference among the five emotions [*Fr*(4) = 27.18, *P* value < 0.001, *Cohen*′*s f*≈0.43], indicating a large effect size. *Post-hoc* power analysis based on the observed effect size indicated sufficient power to detect this effect (*power*≈1.00). Pairwise comparisons (Figure 1) showed that the EDA average for Happiness was significantly higher than Sadness (*P* value < 0.001, *r* = 0.639), Surprise was significantly higher than Anxiety (*P* value < 0.001, *r* = 0.718) and Neutral (*P* value < 0.001, *r* = 0.736).

**Figure 2:**
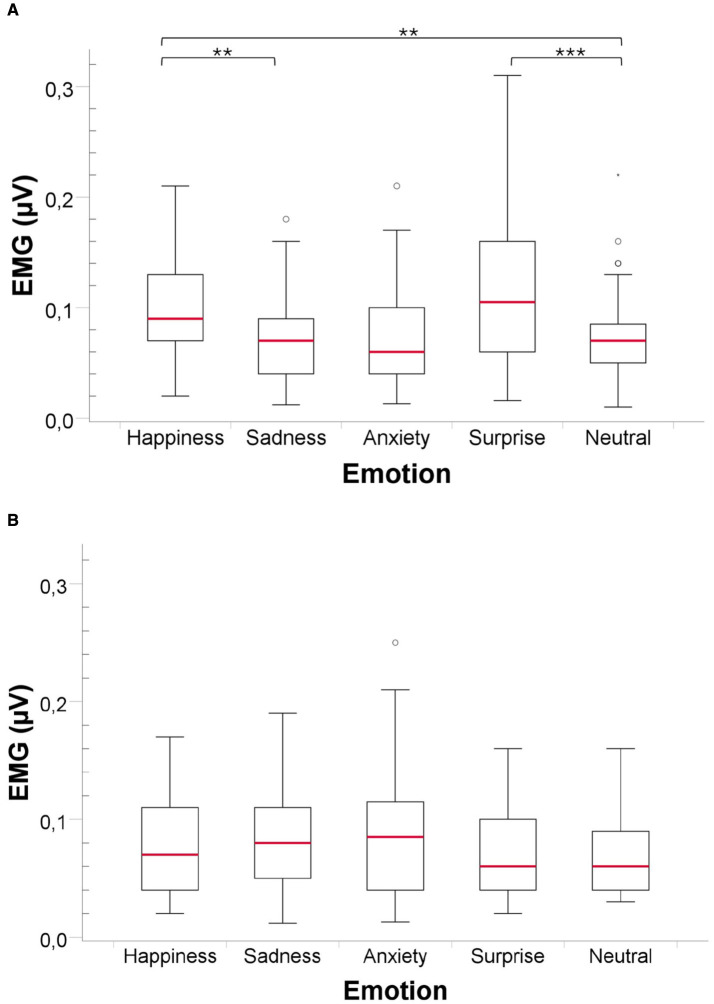
Comparison of EMG activity for the Zygomaticus and Corrugator muscles in response to musical stimuli across all participants from Dataset B, regardless of AD severity. Statistical significance between emotional conditions is indicated by **P_value_ < 0.001 ***P_value_ < 0.001. **(A)** Zygomaticus muscle EMG activity. **(B)** Corrugator muscle EMG activity.

For the EMG zygomaticus, the Friedman test revealed a significant difference among the five emotions [*Fr*(4) = 27.27, *P* value < 0.001, *Cohen*′*s f*≈0.44], indicating a large effect size. *Post-hoc* power analysis indicated sufficient power to detect this effect (*power*≈1.00). Pairwise comparisons (Figure 2A) showed that the EMG level for Happiness was significantly higher than Sadness (*P* value < 0.01, *r* = 0.473), and that Neutral was significantly lower than Happiness (*P* value < 0.01, *r* = 0.520) and Surprise (*P* value < 0.001, *r* = 0.659).

Finally, for the EMG corrugator, the Friedman test revealed a significant difference between the five emotions [*Fr*(4) = 10.54, *P* value < 0.05, *Cohen*′*s f*≈0.27], indicating a small-to-medium effect size. *Post-hoc* power analysis indicated sufficient power to detect this effect (*power*≈0.99). However, pairwise comparisons did not reveal any significant differences across the pairs of emotions (see Figure 2B).

### 3.2 EDA and EMG differences across AD severity levels

We also assessed whether physiological responses differed across AD severity levels (mild, moderate, and severe) for each emotional content in music. As in the previous section, this analysis focused exclusively on AD participants from Dataset B.

**Figure 3:**
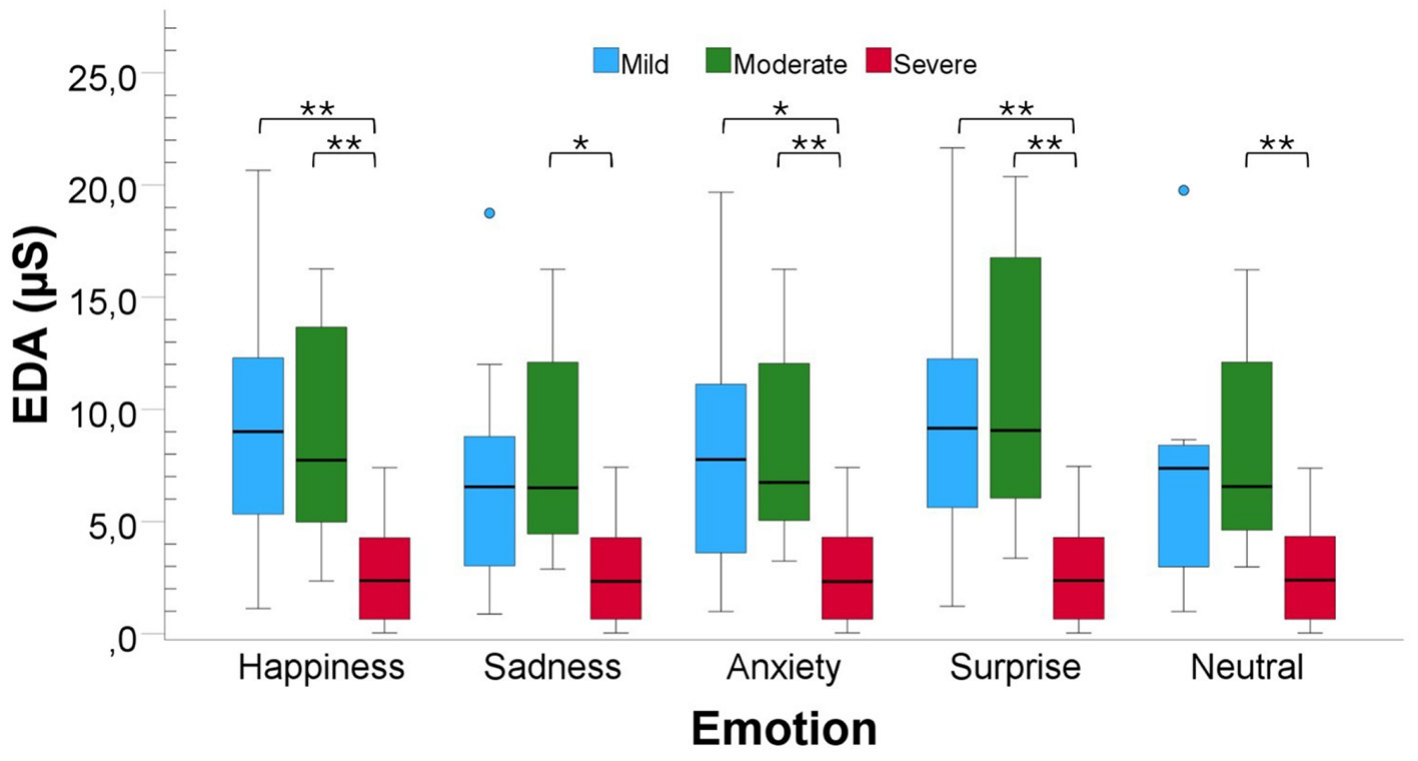
Comparison of EDA responses to musical stimuli across participants with varying AD severity from Dataset B. Bars are color-coded by severity: blue bars for Mild AD, green for Moderate AD, and red for Severe AD. Statistical significance between groups is indicated by *P_value_ < 0.001 **P_value_ < 0.001.

For EDA (Figure 3), the One-Way ANOVA test revealed significant differences between the three AD severity levels for all emotions: Happiness [*F*_(2, 33)_ = 7.75, *P* value < 0.01, *Cohen*′*s f*≈0.69, *power*≈0.95], Sadness [*F*_(2, 33)_ = 5.40, *P* value < 0.01, *Cohen*′*s f*≈0.89, *power*≈1.00], Anxiety [*F*_(2, 33)_ = 6.47, *P* value < 0.01, *Cohen*′*s f*≈0.62, *power*≈0.90], Surprise [*WelchF*_(2, 18.52)_ = 13.15, *P* value < 0.01, *Cohen*′*s f*≈0.72, *power*≈0.97] and Neutral [*F*_(2, 33)_ = 5.52, *P* value < 0.01, *Cohen*′*s f*≈0.58, *power*≈0.85]. Pairwise comparisons revealed significant differences between the Mild and the Severe levels for Happiness (*P* value < 0.01, *r* = 0.60), Anxiety (*P* value < 0.05, *r* = 0.54) and Surprise (*P* value < 0.05, *r* = 0.58), and between the Moderate and Severe levels for Happiness (*P* value < 0.01, *r* = 0.58), Sadness (*P* value < 0.05, *r* = 0.55), Anxiety (*P* value < 0.01, *r* = 0.57), Surprise (*P* value < 0.01, *r* = 0.63) and Neutral (*P* value = 0.01, *r* = 0.57).

**Figure 4:**
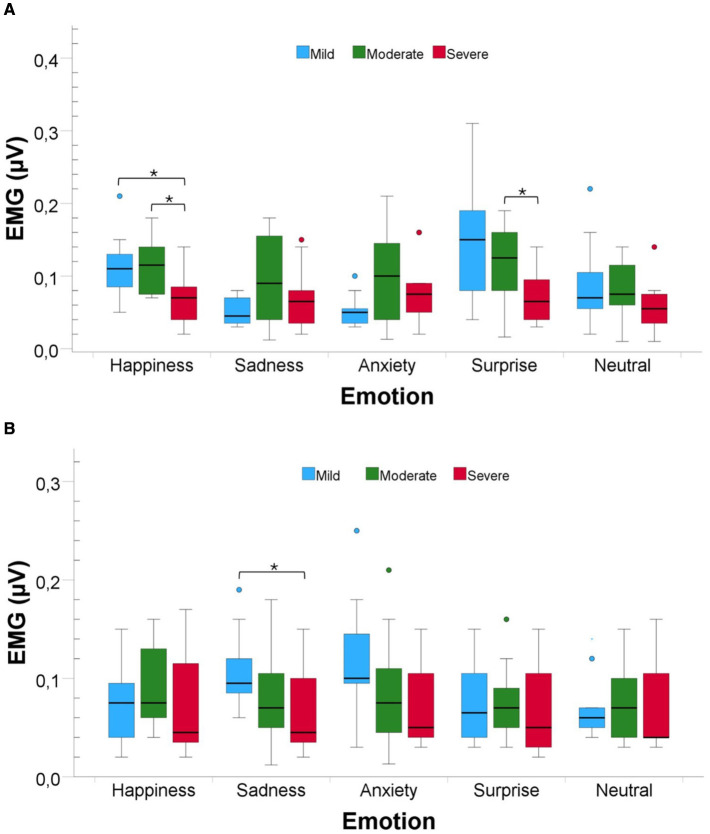
Comparison of EMG activity for the Zygomaticus and Corrugator muscles in response to musical stimuli across participants with varying AD severity from Dataset B. Bars are color-coded by severity: blue bars for Mild AD, green for Moderate AD, and red for Severe AD. Statistical significance between groups is indicated by **P_value_ < 0.001. **(A)** Zygomaticus muscle EMG activity. **(B)** Corrugator muscle EMG activity.

Regarding the EMG zygomaticus muscle (Figure 4A), the one-way ANOVA test revealed significant differences between the three AD levels for Happiness [*F*_(2, 33)_ = 5.08, *P* value < 0.05, *Cohen*′*s f*≈0.56, *power*≈0.83] and surprise [*F*_(2, 33)_ = 4.51, *P* value < 0.05, *Cohen*′*s f*≈0.53, *power*≈0.78]. Pairwise comparisons revealed that for Happiness, there was a significant difference between the mild and severe (*P* value < 0.05, *r* = 0.50) levels and between the moderate and severe levels (*P* value < 0.05, *r* = 0.52). For surprise, we only found a significant difference between the mild and severe levels (*P* value < 0.05, *r* = 0.53). No significant results were found between the three severity levels for the remaining emotions: Sadness, Anxiety and Neutral.

Finally, for the EMG corrugator muscle (Figure 4B), the Kruskal–Wallis test only revealed a significant difference between the three severity levels for Sadness [*H*_(2)_ = 6.48, *P* value < 0.05, *Cohen*′*s f*≈0.40, *power*≈0.53]. The pairwise comparison revealed a significant difference between the Mild and Severe levels of AD severity (*P* value < 0.05, *r* = 0.42).

### 3.3 Classification of emotional content in music

**Figure 5:**
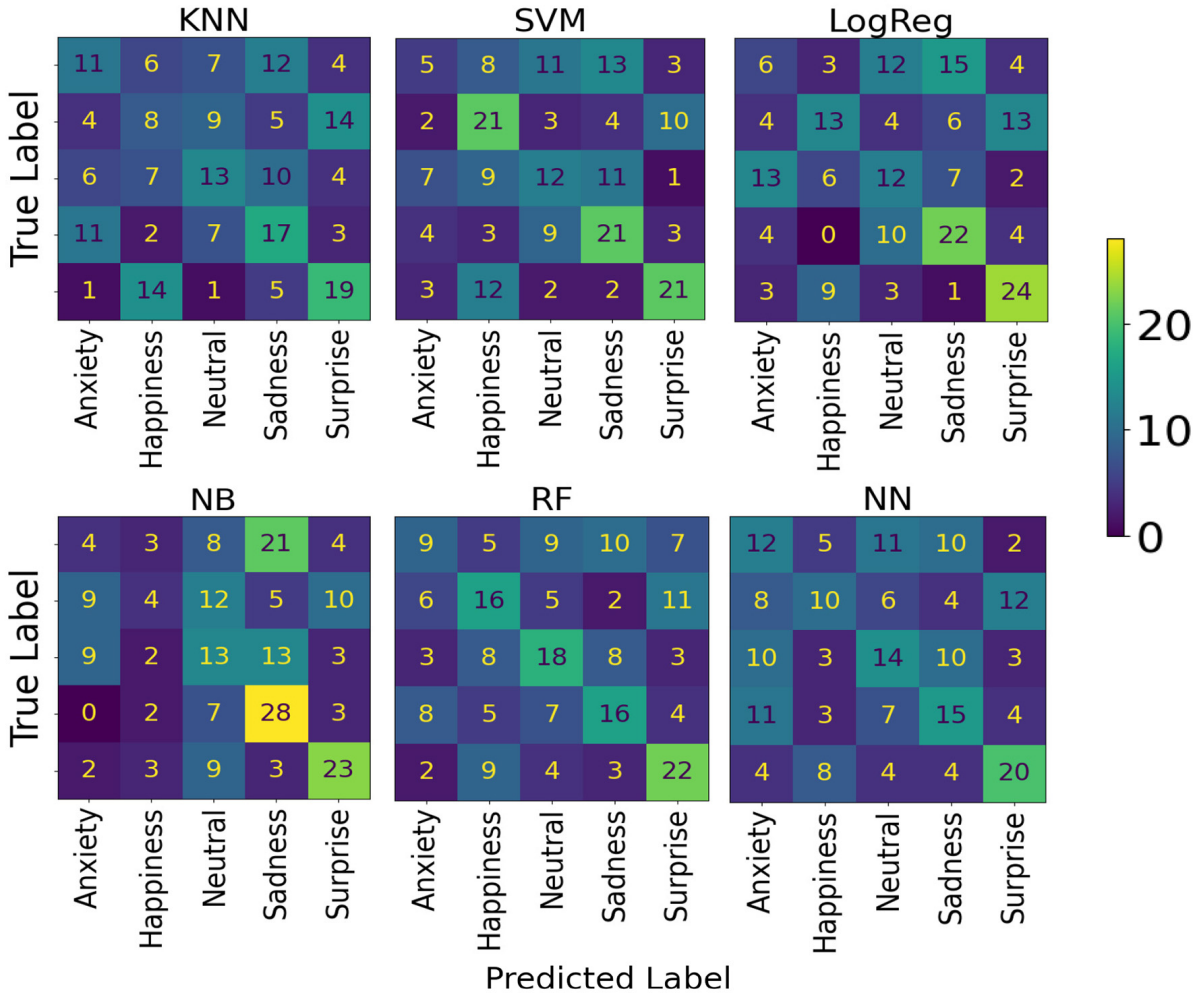
Model confusion matrices for classification of emotional content in music, using Dataset A.

The results obtained for classifying emotional content in music (happiness, neutral, sadness, and surprise) using Dataset A are shown in Table 1. The model with the highest accuracy in predicting the emotional content of music was the RF, with an average accuracy of 40.50% (95% CI: 34.11–46.89), followed by the SVM, with an average accuracy of 40.00% (95% CI: 33.21–46.79). The confusion matrices for these models are shown in Figure 5.

**Table 1:** Model accuracy comparison for classification of the emotional content in music, using Dataset A.

**Classifier**	**Accuracy (%)**	**95% CI**	**Precision (%)**	**Recall (%)**	**F1-Score (%)**
KNN	34.00	27.99–40.01	61.08	34.00	25.55
SVM	40.00	33.21–46.79	69.37	40.00	30.10
NB	36.00	30.54–41.46	66.08	36.00	26.72
LogReg	38.50	32.82–44.18	67.60	38.50	28.20
**RF**	**40.50**	**34.11–46.89**	**68.92**	**40.50**	**30.25**
NN	35.50	27.77–42.23	61.79	35.50	27.50

The highlighted row represents the model with the best accuracy.

**Figure 6:**
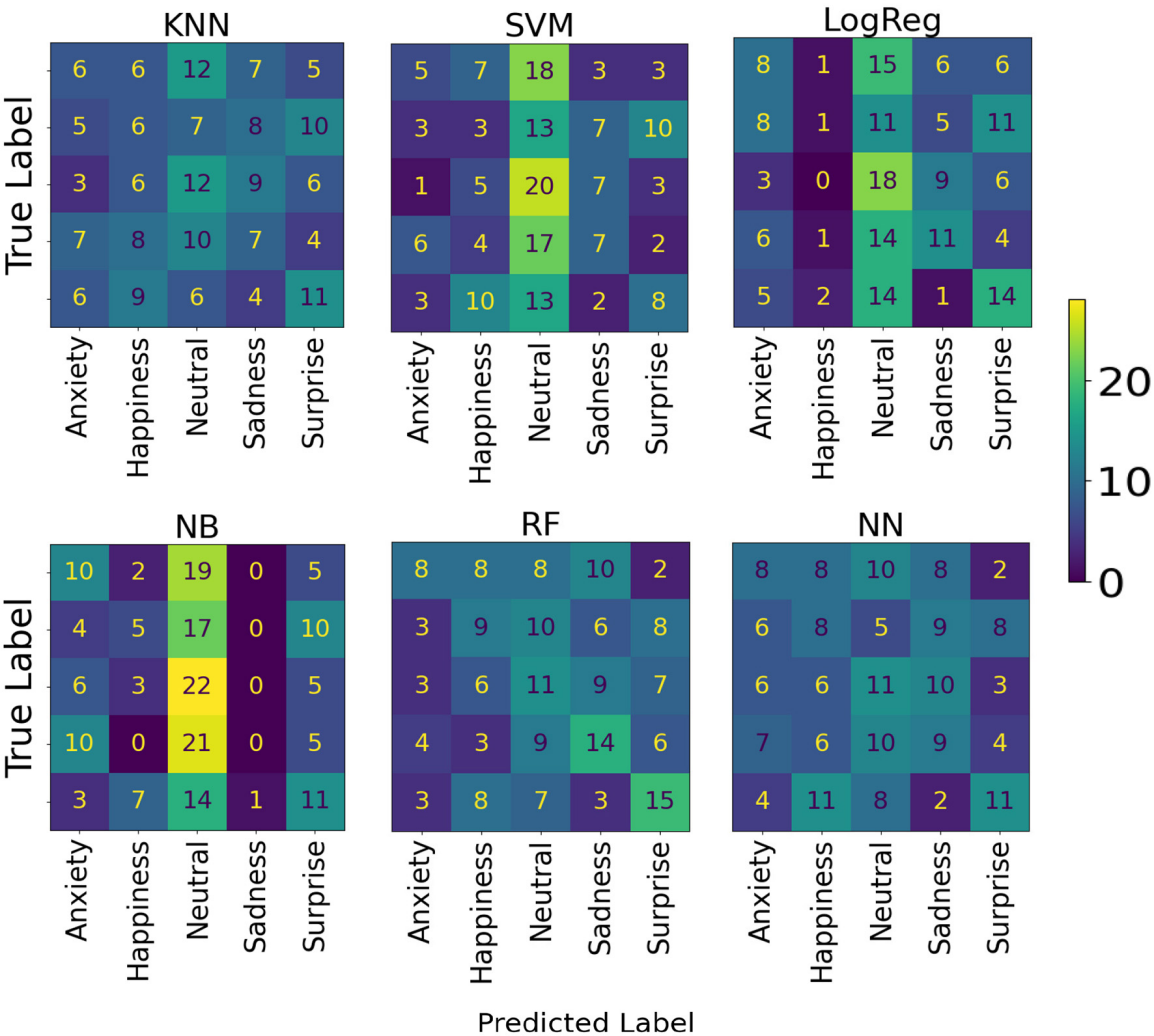
Model confusion matrices for predicting emotional content in music, using Dataset B.

For Dataset B, the results obtained are shown in Table 2. The model with the best accuracy for predicting emotional content in music among AD patients was RF, with an average accuracy of 32.22% (95% CI: 25.50–38.95). The models' confusion matrices are shown in Figure 6.

**Table 2:** Model accuracy comparison for classification of the emotional content in music using Dataset B.

**Classifier**	**Accuracy (%)**	**95% CI**	**Precision (%)**	**Recall (%)**	**F1-Score (%)**
KNN	17.22	11.82–22.62	60.98	17.22	9.39
SVM	31.67	24.01–39.32	74.34	31.67	21.39
NB	26.67	20.83–32.50	74.38	26.67	15.09
LogReg	28.33	22.64–34.02	74.18	28.33	17.02
**RF**	**32.22**	**25.50–38.95**	**66.85**	**32.22**	**22.07**
NN	21.11	16.55–25.67	63.64	21.11	12.57

The highlighted row represents the model with the best accuracy.

### 3.4 Classification healthy vs. AD participants

**Figure 7:**
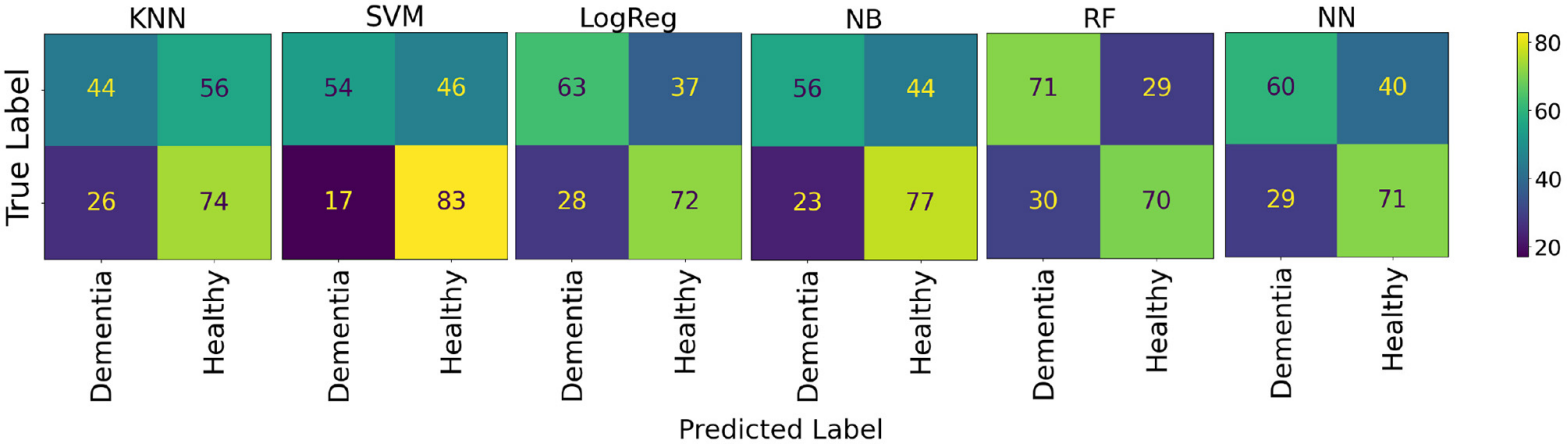
Model confusion matrices for classification between healthy and AD participants.

The results of distinguishing between healthy and AD participants are shown in Table 3. The RF model achieved the highest average accuracy of 70.50% (95% CI: 57.55–83.45) in distinguishing between healthy and AD participants. The confusion matrices for these models are shown in Figure 7.

**Table 3:** Model accuracy for classification between healthy and AD participants.

**Classifier**	**Accuracy (%)**	**95% CI**	**Precision (%)**	**Recall (%)**	**F1-Score (%)**
KNN	59.00	47.24–70.76	100.00	59.00	66.70
SVM	68.50	55.15–81.85	100.00	68.50	71.60
NB	66.50	53.51–79.49	100.00	66.50	70.46
LogReg	67.50	53.71–81.29	100.00	67.50	69.90
**RF**	**70.50**	**57.55–83.45**	**100.00**	**70.50**	**73.55**
NN	65.50	52.30–78.70	100.00	65.50	69.28

The highlighted row represents the model with the best accuracy.

### 3.5 Classification of AD severity

**Figure 8:**
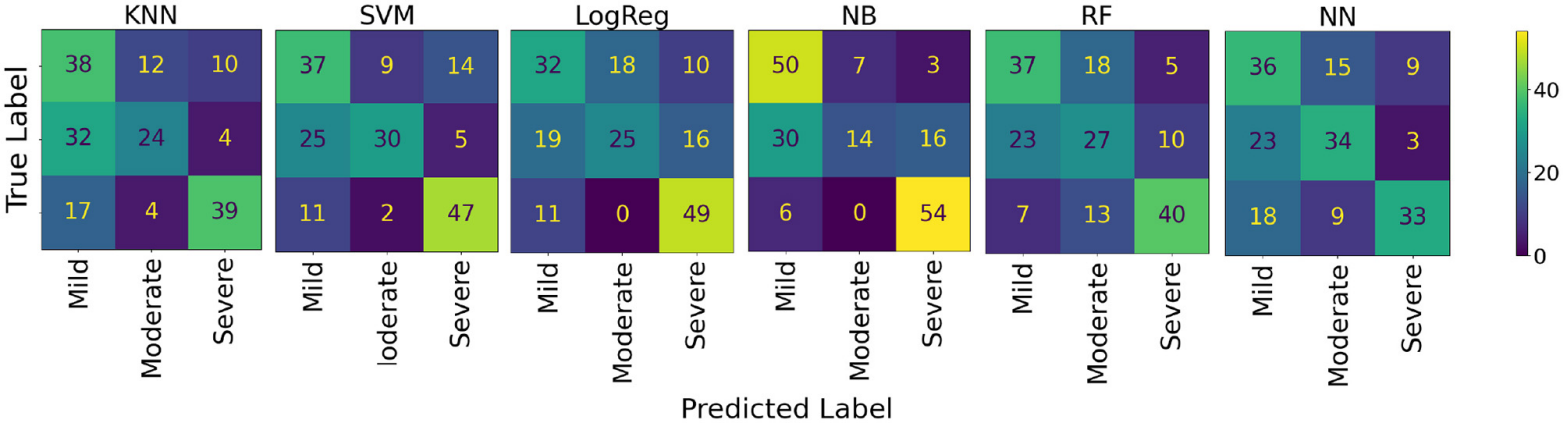
Model confusion matrices for classifying AD severity: mild, moderate and severe.

The results obtained to assess AD severity among AD participants are shown in Table 4. The NB was the best model for distinguishing between mild, moderate, and severe dementia, with an average accuracy of 65.56% (95% CI: 50.41–80.70). The confusion matrices for these models are shown in Figure 8.

**Table 4:** Model accuracy comparison for predicting AD severity: mild, moderate and severe.

**Classifier**	**Accuracy (%)**	**95% CI**	**Precision (%)**	**Recall (%)**	**F1-Score (%)**
KNN	56.11	43.29–68.93	100.00	56.11	62.99
SVM	63.33	50.14–76.52	100.00	63.33	69.07
**NB**	**65.56**	**50.41–80.70**	**100.00**	**65.56**	**67.56**
LogReg	58.89	44.07–73.71	100.00	58.89	62.54
RF	57.78	43.98–71.58	100.00	57.78	63.34
NN	57.22	43.20–71.25	100.00	57.22	62.36

The highlighted row represents the model with the best accuracy.

## 4 Discussion

This study evaluated the emotional responses of AD patients while listening to musical excerpts across different severity levels (mild, moderate, severe), using EDA to quantify arousal and facial EMG to quantify valence. We also developed ML models to predict the emotional content in music, distinguish healthy from AD participants, and classify AD severity.

### 4.1 Physiological responses to emotional content in music

Our results indicate that individuals with AD retain partially preserved physiological responses to emotionally evocative music. EDA increased during excerpts designed to elicit happiness and surprise, whereas zygomaticus activity was elevated during happiness relative to sadness and neutral conditions. These findings align with prior research showing preserved emotional responsiveness in AD (Walker et al., 2021), demonstrating that even passive music listening can evoke measurable arousal and positive valence.

Facial EMG analysis confirmed that zygomaticus activity, indicative of positive valence, was significantly elevated during happiness and surprise, consistent with activation of reward and autobiographical memory networks (Cuddy et al., 2015). Corrugator activity, associated with negative valence, showed non-significant increases during anxiety, reflecting generalized negative affect or reduced emotional specificity (Burton and Kaszniak, 2006; Fuentes-Sánchez et al., 2021). These results support the notion that emotional processing, particularly for positive stimuli, remains partially intact in AD despite cognitive decline.

Stratification by AD severity revealed a decline in physiological engagement. Participants with severe AD exhibited lower EDA across all conditions and reduced zygomaticus activation, especially for happiness and surprise. This decline likely reflects neuropathological changes, including *Aβ* accumulation and neuronal atrophy in frontal, basal ganglia, and brainstem regions (Barradas et al., 2021). Corrugator activation during sadness was also lower in severe compared to mild patients, indicating reduced sensitivity to negative stimuli and potential frontal lobe dysfunction (Burton and Kaszniak, 2006). Non-significant increases in zygomaticus activity during sadness and anxiety in moderate and severe patients may reflect dysregulated motor control and incongruent affective displays (Walker et al., 2021; Burton and Kaszniak, 2006; Sun et al., 2021). These findings highlight that while emotional reactivity becomes less differentiated and blunted with disease progression, residual affective processing persists. Notably, the reduced differentiation observed for negative emotions is also reflected in the ML classification results. While positive emotions, such as happiness and surprise, were more reliably distinguished, sadness and anxiety elicited subtler and more variable physiological responses across AD severity levels, making them more challenging for the models to classify. This pattern aligns with known neuropathological effects on cortical and limbic regions involved in processing negative affect, including the prefrontal cortex and amygdala, whose progressive degeneration in AD diminishes the specificity of responses to aversive or negative musical cues.

Music-evoked physiological responses, such as EDA and EMG, arise from a coordinated interplay between limbic, subcortical, and brainstem circuits, which are progressively disrupted in AD. The amygdala plays a central role in emotional valence, driving sympathetic arousal reflected in EDA responses (Koelsch, 2020). As AD progresses, amygdala degeneration may contribute to the blunted arousal differentiation observed in severe stages. The hippocampus, which links music to autobiographical and emotional memories, supports activation of the zygomaticus muscle during positive and familiar excerpts (Jacobsen et al., 2015; Cuddy et al., 2015). Its relative preservation in early AD likely underlies the sustained positive valence responses found in mild patients. Additionally, brainstem structures mediate rapid, reflexive reactions to sudden acoustic changes –such as pitch, rhythm, or tempo–and are comparatively spared in the early stages of the disease (Koelsch, 2020). This preservation may explain why even patients with severe AD still exhibit residual EDA increases in response to surprising or attention-grabbing musical cues.

These stage-specific physiological patterns provide insights into potential applications for diagnostic staging and targeted interventions. In mild stages, preserved autonomic and facial reactivity to emotionally positive music suggests that such stimuli could be leveraged to detect early alterations in emotional processing and therapeutically to enhance mood, attention, and autobiographical recall. As AD progresses, the blunted differentiation in physiological responses observed in moderate and severe patients reflects diminished cortical and limbic engagement, indicating the need for simpler musical interventions that target residual subcortical mechanisms. These differential signatures could inform stratified music-based interventions and support stage-sensitive clinical decisions.

Taken together, these observations suggest that the physiological patterns observed in this study reflect a hierarchical degradation of emotion-related neural pathways in AD, characterized by relatively intact subcortical and brainstem reflexes, partially preserved limbic responses, and declining cortical modulation as the disease progresses. As emotional reactivity becomes less differentiated, residual affective processing persists, but the individual's remaining cognitive resources shape its expression. Prior studies have shown that memory, attention, and executive function modulate emotional recognition and engagement with music in AD (Jacobsen et al., 2015). Therefore, tailoring music-based interventions to both cognitive and emotional capacities may optimize responsiveness and therapeutic benefit, particularly as cognitive decline affects how emotions are perceived and expressed in later stages of the disease.

### 4.2 Classification models

Our ML models trained on physiological data demonstrated above-chance performance across all classification tasks. The Random Forest classifier achieved 70.50% accuracy (95% CI: 57.55–83.45) for distinguishing healthy and AD participants, and the Naïve Bayes classifier achieved 65.56% accuracy (95% CI: 50.41–80.70) for AD severity classification. Classification of musical emotions was more challenging in AD participants [32.22% (95% CI: 25.50–38.95)] compared to healthy controls [40.50% (95% CI: 34.11–46.89)], both of them with the Random Forest classifier, reflecting inter-subject variability in affective responses (Lima et al., 2024).

The observed differences in model performance likely reflect each algorithm's ability to handle the complex, nonlinear dynamics of physiological data. The Random Forest classifier achieved the best performance in distinguishing between healthy and AD participants and in emotion classification across both datasets, likely due to its ensemble learning structure, which effectively models nonlinear interactions among physiological signals in small sample sizes. In contrast, Naïve Bayes performed best for AD severity classification, suggesting that its probabilistic framework handled noisy, overlapping data distributions more effectively in small samples. More complex models, such as SVMs and NNs, performed less consistently due to their higher variance and sensitivity to the small dataset size. Conversely, KNN and LogReg also demonstrated limited generalizability, suggesting that distance-based and linear classifiers may not effectively capture the nonlinear and multimodal interactions in physiological responses. These findings underscore the importance of model selection in achieving a balance between complexity and generalization, particularly when working with heterogeneous physiological data from clinical populations.

This study is among the first studies to integrate EDA and facial EMG into ML pipelines for AD classification (Zhang C. et al., 2024). Our results suggest that physiological measures can provide objective, complementary information about emotional processing in AD. To contextualize these results, similar accuracy ranges have been reported in biomarker-based ML studies. For instance, cerebrospinal fluid (CSF) biomarkers achieved 75%–85% accuracy in staging AD (Tiwari et al., 2024), blood biomarker models reached 80%–90% accuracy in differentiating neurodegenerative diseases (Kelly et al., 2023), and multimodal neuroimaging approaches combining structural MRI and resting-state MEG attained up to 93.5% accuracy (Liu et al., 2024; Blanco et al., 2023). Although these methods rely on complex or invasive data, the present study highlights that music-evoked physiological responses provide a non-invasive, low-cost, and ecologically valid alternative. While our physiological markers do not constitute standalone biomarkers and should not be interpreted as definitive diagnostic tools, they offer added value for research into emotional responsiveness and the early detection of disease.

### 4.3 Clinical relevance and limitations

Our classification performance provided meaningful insights as a complementary tool for early-stage AD screening or monitoring, rather than a standalone diagnostic method. Unlike traditional cognitive assessments, such as the MMSE or Montreal Cognitive Assessment (MoCA), which rely on subjective performance and can be influenced by education, language, and fatigue, physiological measures capture objective responses to emotional stimuli. Similarly, neuroimaging methods provide information on brain atrophy or *Aβ* accumulation but are costly, resource-intensive, and not easily repeated. In contrast, EDA and facial EMG recording are non-invasive, low-cost methods that can be administered repeatedly, even in non-verbal or severely impaired patients. Integrating these physiological responses with traditional assessments may enhance early detection and enable continuous, objective monitoring of disease progression.

However, the practical implementation of this approach in real-world clinical settings presents challenges. In this study, we used the Biosignalsplux wearable device with physiological electrodes, the OpenSignals software, and musical excerpts from BRECVEMA to elicit emotional responses. Although these tools are relatively low-cost and non-invasive compared to neuroimaging, consistent measurement requires trained personnel to ensure high-quality data acquisition and reliable results. Personnel must correctly place EDA and facial EMG electrodes and visually inspect the signal quality during baseline and musical excerpts. Consistency in baseline recordings, stimulus administration, and environmental conditions must be maintained to allow accurate comparisons across participants. Proper training and standardized procedures are essential to guarantee reproducible measurements and to address logistical and operational challenges before physiological measures can be routinely integrated into clinical practice.

While these findings highlight the potential clinical value of physiological measures as complementary to existing AD assessment protocols, several methodological limitations must be considered when interpreting these results.

We acknowledge that, despite above-chance performance, these modest accuracies imply misclassification rates, including potential false positives and false negatives, which must be carefully considered when evaluating practical clinical applications. Nevertheless, this classification performance provides meaningful insights as a complementary tool for early-stage screening or monitoring, rather than as a standalone diagnostic method. Recruitment of participants at specific stages of AD was challenging due to the geographically limited population, and sample sizes, particularly for Dataset B, were relatively small, limiting statistical power and generalizability. Additionally, although both datasets A and B were collected using the same experimental protocol, differences in cohort characteristics could introduce subtle bias. To mitigate this, Dataset A was used solely for classification tasks involving healthy participants, while Dataset B was used independently to analyze AD patients and severity-specific responses. Importantly, the datasets were not merged, thereby ensuring consistency across analyses.

To minimize overly optimistic performance estimates, we employed LOSO cross-validation and averaged metrics across all folds, ensuring that model performance reflects predictions on unseen participants. While this approach helps mitigate overfitting, modest sample sizes inherently limit the robustness of ML models. Larger datasets from a single cohort, combined with multimodal approaches that integrate additional physiological signals, cognitive assessment tools, and neuroimaging data, could enhance model generalizability and predictive accuracy. Moreover, the current study used a limited set of nine features across the three physiological signals. While these features captured meaningful differences in emotional responses, incorporating additional features could provide a more comprehensive characterization of physiological reactions to music, potentially improving classification performance. These considerations are relevant for translating findings into clinically meaningful applications.

Several potential confounding factors may have influenced physiological responses and classification performance. All participants were taking anti-dementia medications, which could affect emotional processing and physiological reactivity. While participants with hearing problems were excluded, subclinical hearing impairments may have influenced responses to the musical excerpts. Other comorbid conditions common in elderly populations, such as cardiovascular or metabolic disorders, could also have affected EDA and facial EMG signals. We controlled for major confounders where possible, but residual effects cannot be entirely ruled out. Although the effects of medication were not directly controlled in this study, the observed stage-specific differences in EDA and EMG remain consistent with known neuropathological progression; however, they should be interpreted in consideration of potential pharmacological modulation.

Additional factors related to the musical stimuli and cultural context may also have influenced our results. We did not assess participants' musical preferences, which research shows significantly impacts emotional and physiological responses to music, particularly in AD populations. Although the stimuli were validated with Portuguese elderly participants (Barradas et al., 2021), our sample's cultural homogeneity limits the generalizability of our findings to other populations. Notably, only the Happiness condition included familiar music that was explicitly selected for Portuguese participants. In contrast, the remaining conditions deliberately employed unfamiliar excerpts to ensure that emotional mechanisms, rather than familiarity, drove responses.

Future studies should systematically account for medications, sensory impairments, and comorbidities to ensure that observed physiological differences are attributable to AD-related changes rather than extraneous factors. Moreover, clinical applications should consider individual musical preferences and cultural backgrounds when developing personalized music-based interventions for AD patients.

Overall, emotionally evocative music can elicit measurable arousal and valence responses, even in moderate-to-severe AD. Positive musical stimuli, such as happiness and surprise, produce the most consistent physiological responses, while negative emotions are less differentiated. Emotional responsiveness declines with disease progression, consistent with neuropathology and previous literature (Cuddy et al., 2015; Walker et al., 2021; Burton and Kaszniak, 2006).

In conclusion, individuals with AD exhibit partially preserved physiological responses to emotional music, especially to positive stimuli, although these responses diminish with disease severity. Machine learning models applied using EDA and facial EMG successfully differentiated between healthy and AD participants, capturing patterns related to disease severity and indicating the potential utility of emotional physiology as a complementary diagnostic tool.

## Data Availability

The raw data supporting the conclusions of this article will be made available by the authors, without undue reservation.
